# Effect of an alkyl spacer on the morphology and internalization of MUC1 aptamer‐naphthalimide amphiphiles for targeting and imaging triple negative breast cancer cells

**DOI:** 10.1002/btm2.10194

**Published:** 2020-11-10

**Authors:** Huihui Kuang, Zachary Schneiderman, Ahmed M. Shabana, Gabriella C. Russo, Jun Guo, Denis Wirtz, Efrosini Kokkoli

**Affiliations:** ^1^ Institute for NanoBioTechnology Johns Hopkins University Baltimore Maryland USA; ^2^ Department of Chemical and Biomolecular Engineering Johns Hopkins University Baltimore Maryland USA; ^3^ Department of Pharmaceutics and Industrial Pharmacy, Faculty of Pharmacy Cairo University Cairo Egypt

**Keywords:** aptamer nanofibers, effect of spacer on self‐assembly, nanoparticles, ssDNA aptamer‐amphiphiles, targeted drug delivery, targeting MUC1

## Abstract

Despite decades of research, there are few targeted treatment options available for triple negative breast cancer (TNBC), leaving chemotherapy, and radiation treatment regimes with poor response and high toxicity. Herein aptamer‐amphiphiles were synthesized which selectively bind to the mucin‐1 (MUC1) glycoprotein that is overexpressed in TNBC cells. These amphiphiles have a fluorescent tail (1,8‐naphthalimide or 4‐nitro‐1,8‐naphthalimide) which enables self‐assembly of the amphiphiles and allows for easy visualization without the requirement for further conjugation of a fluorophore. Interestingly, the length of the alkyl spacer (C_4_ or C_12_) between the aptamer and tail was shown to influence the morphology of the self‐assembled structure, and thus its ability to internalize into the TNBC cells. While both the MUC1 aptamer‐C_4_‐napthalimide spherical micelles and the MUC1 aptamer‐C_12_‐napthalimide long cylindrical micelles showed internalization into MDA‐MB‐468 TNBC cells but not the noncancerous MCF‐10A breast cells, the cylindrical micelles showed greatly enhanced internalization into the MDA‐MB‐468 cells. Similar patterns of enhanced binding and internalization were observed between the MUC1 aptamer‐C_12_‐napthalimide cylindrical micelles and SUM159 and MDA‐MB‐231 TNBC cells. The MUC1 aptamer cylindrical micelles were not toxic to the cells, and when used to deliver doxorubicin to the TNBC cells, were shown to be as cytotoxic as free doxorubicin. Moreover, a pharmacokinetic study in mice showed a prolonged systemic circulation time of the MUC1 aptamer cylindrical micelles. There was a 4.6‐fold increase in the elimination half‐life of the aptamer cylindrical micelles, and their clearance decreased 10‐fold compared to the MUC1 aptamer spherical micelles. Thus, the MUC1 aptamer‐C_12_‐napthalimide nanofibers represent a promising vehicle that could be used for easy visualization and targeted delivery of therapeutic loads to TNBC cells.

## Graphical abstract

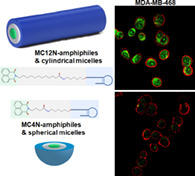

## 1 INTRODUCTION

Breast cancer is a leading cause of cancer related mortalities, and TNBC represents approximately 15–20% of all invasive types of breast cancer.^1^ TNBC, characterized by its lack of estrogen and progesterone receptors and normal HER2 expression, has proven extremely difficult to treat, even after over a decade of research.^2^ Due to the lack of many primary therapeutic targets, there are few targeted therapy options available, leaving only surgery, chemotherapeutics, and radiation therapy as viable treatment options.^2^, ^3^, ^4^, ^5^ This route tends to be less effective with more pronounced side effects. As such, discovery of targeted delivery strategies is critical to improving breast cancer patient outcomes and experiences.^6^, ^7^ One potential target is the transmembrane glycoprotein MUC1, which has been the subject of significant clinical research for both antibody‐based treatments and vaccines. Not only is MUC1 known to be overexpressed in most carcinomas, including ~90% of breast cancer tumors, but it is linked to immune evasion, cancer progression, and metastasis.^8^, ^9^, ^10^, ^11^, ^12^, ^13^ Incomplete O‐linked glycosylation (underglycosylation) of MUC1 expressed on the surface of epithelial cancer cells results in exposed peptide epitopes and shortened carbohydrate chains, such as the Thomsen‐nouvell (Tn) antigen.^14^, ^15^, ^16^ Tn is a single N‐acetylgalactosamine (GalNAc) added to a serine or threonine of a protein, and represents the first step of an O‐linked glycosylation pathway. Thus, underglycosylation of MUC1 on cancer cells can generate glycans truncated at the initial GalNAc (Tn), leaving them exposed for targeting. The MUC1 glycoprotein typically internalizes via clathrin‐mediated endocytosis or macropinocytosis and to a lesser extent via caveolae‐mediated endocytosis, thus enabling cell internalization of targeted nanoparticles bound to it.^17^, ^18^, ^19^, ^20^

In this study, a single‐stranded DNA (ssDNA) aptamer was used to target MUC1 which specifically binds to the GalNAc regions of the protein. More importantly, the MUC1 aptamer was shown to bind to MUC1+ cancer cells, but not MUC1− cells or even MUC1+ normal human primary epithelial cells displaying fully glycosylated MUC1.^14^ Thus, this aptamer, targeting the underglycosylated MUC1 overexpressed on the surface of cancer cells, presents a promising targeting ligand for the design of a novel delivery system aimed at TNBC.

While aptamers are often conjugated to other drug delivery systems and molecular diagnostic tools such as quantum dots or peptide, polymeric, or Au nanoparticles, the ssDNA itself can be induced to self‐assemble into nanostructures via conjugation of a hydrophobic tail.^21^, ^22^, ^23^, ^24^, ^25^, ^26^ Direct conjugation of ssDNA aptamers to hydrophobic tails largely generates spherical micelles, however the inclusion of an alkyl spacer between a hydrophobic di‐alkyl tail and hydrophilic ssDNA headgroup induced more complex and interesting morphologies.^27^, ^28^, ^29^, ^30^ These may in turn be able to impact the ability of a delivery system to associate with cells given the widely reported impact of size and shape on nanoparticle delivery.^31^, ^32^, ^33^, ^34^

The hydrophobic tail of the amphiphile promotes self‐assembly, and can also serve other functions such as enabling visualization or delivering a therapeutic load.^35^, ^36^ In this work, 1,8‐naphthalimide (or 4‐nitro‐1,8‐naphthalimide for enhanced fluorescence) was chosen as an example of a hydrophobic fluorophore that can serve as a tail for the design of the MUC1 aptamer‐amphiphile, as it has been used in a wide variety of in vitro and in vivo studies for imaging, and shows no cytotoxicity on its own.^37^ Altogether, this makes it a suitable choice for the amphiphile tail. Incorporation of a spacer between the hydrophobic tail and hydrophilic headgroup has been shown to play a vital role on the assembly and binding properties of both ssDNA‐amphiphiles and peptide‐amphiphiles.^27^, ^30^, ^38^ While a myriad of spacers of varying lengths and types could have been used, in this work C_4_ and C_12_ spacers were utilized as previous work has demonstrated that alkyl spacers can promote the assembly of ssDNA supramolecular architectures.^29^, ^30^ After the synthesis of different MUC1 aptamer‐amphiphiles, circular dichroism (CD) spectroscopy was used to evaluate the effect of the tail conjugation on the aptamer's secondary structure, and their assembled structures were identified via cryogenic transmission electron microscopy (cryo‐TEM). Flow cytometry and confocal microscopy were then utilized to examine the cell association and internalization ability of both MUC1 aptamer‐amphiphile structures in representative TNBC cells, MDA‐MB‐468, SUM159, and MDA‐MB‐231, and a noncancerous breast cell line, MCF‐10A. Finally, the ability of the MUC1 aptamer‐amphiphile nanoparticles to deliver a therapeutic load, such as doxorubicin (DOX), to TNBC cells in vitro was evaluated, along with their pharmacokinetic properties in mice.

## 2 RESULTS AND DISCUSSION

**SCHEME 1:**
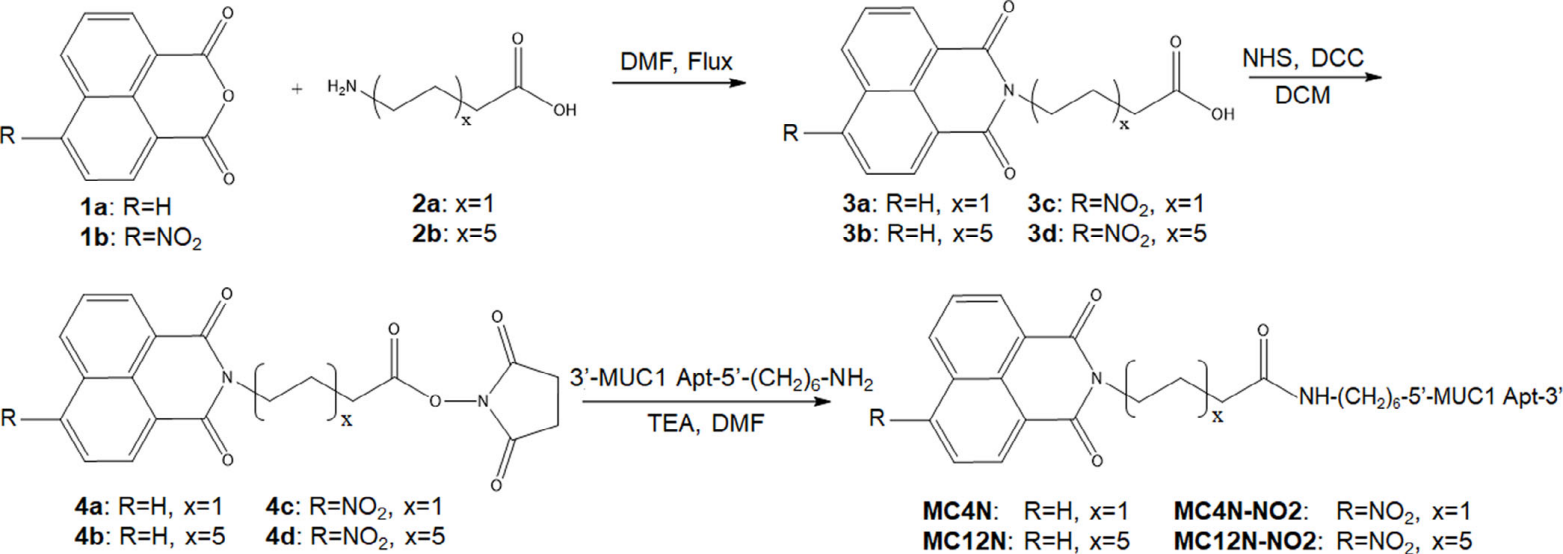
Synthesis of MUC1 aptamer‐naphthalimide amphiphiles

**FIGURE 1:**
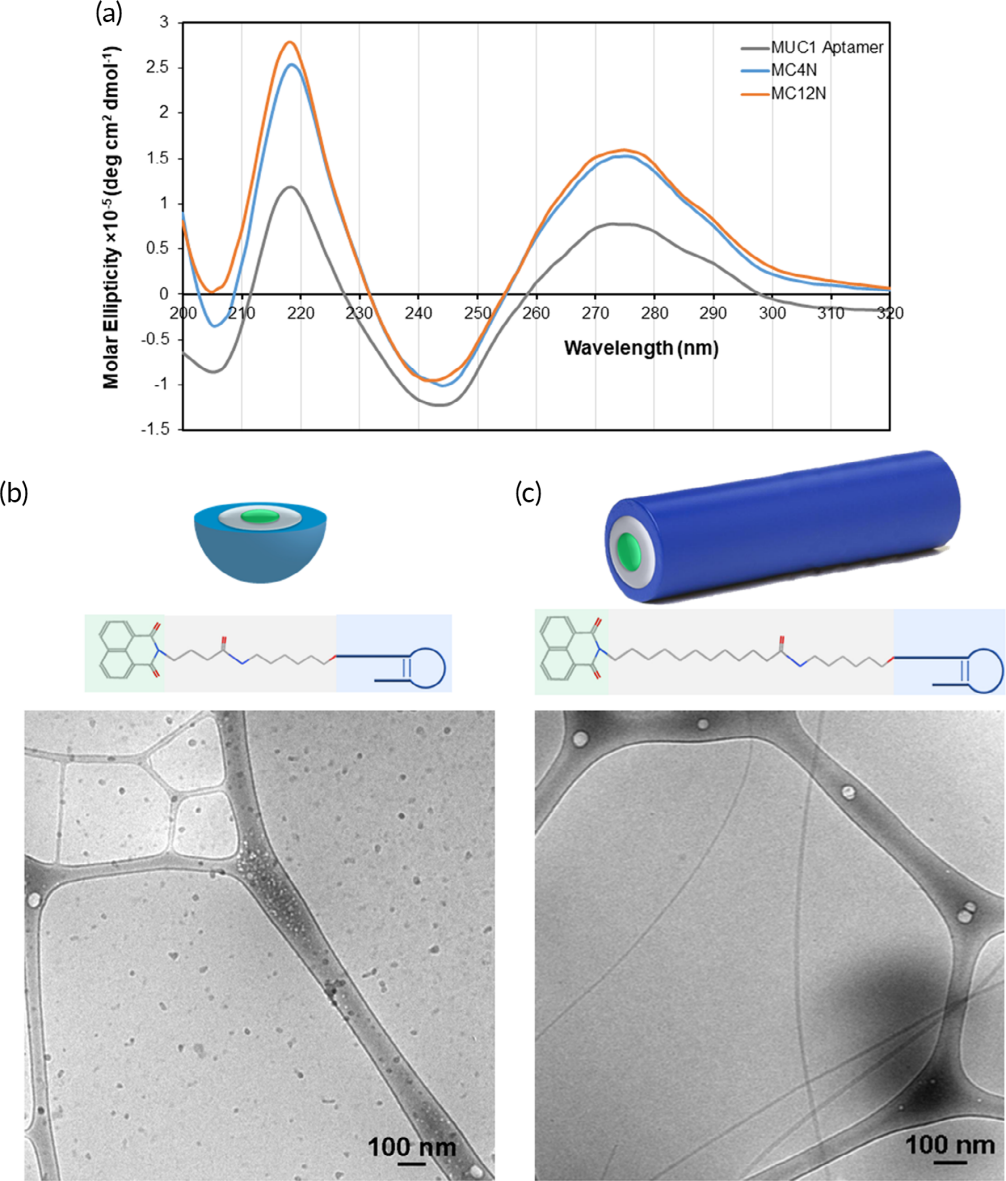
Characterization of MC4N and MC12N nanoparticles. (a) Circular dichroism (CD) spectra of the MUC1 aptamer, MC4N‐ and MC12N‐amphiphiles in Milli‐Q water. Schematic representation of the self‐assembled nanoparticles (MUC1 aptamer is shown in blue, C_4_ and C_12_ spacer in gray, and 1,8‐naphthalimide in green) and cryogenic transmission electron microscopy (cryo‐TEM) images (bottom) of (b) spherical micelles formed from MC4N aptamer‐amphiphiles, and (c) cylindrical micelles formed from MC12N aptamer‐amphiphiles

The MUC1 aptamer‐amphiphiles were synthesized as described in Scheme 1. Successful synthesis of the tail‐spacer molecules was verified by ^1^H NMR (Figures S1 and S2), and liquid chromatography‐mass spectrometry was used for the verification of the masses of the MUC1 aptamer‐amphiphiles. All initial experiments were performed with the MC4N and MC12N aptamer‐amphiphiles, and the ssDNA in all nanoparticles of this study is the aptamer sequence, which is typical for aptamer micelles.^39^, ^40^, ^41^, ^42^ Conjugation of the spacer and aptamer did not inhibit the fluorescence of 1,8‐naphthalimide. Fluorescence spectroscopy confirmed that both the MC4N and MC12N aptamer‐amphiphiles exhibited a maximum excitation and emission wavelength of 345 and 385 nm, respectively (Figure S3). The absorbance and fluorescence of 1,8‐naphthalic anhydride is also shown for comparison. The secondary structure of the aptamer and aptamer‐amphiphiles was evaluated via CD spectroscopy (Figure 1(a)), which showed that the MUC1 aptamer, MC4N and MC12N aptamer‐amphiphiles all had a peak maximum at 275 nm, and a minimum at ~244 nm, consistent with a stem‐loop secondary structure that has been observed before for the MUC1 aptamer.^30^ Therefore, conjugation of the MUC1 aptamer to the tail‐spacer molecules did not affect the secondary structure of MUC1 aptamer. Cryo‐TEM was used to characterize the self‐assembled structures formed by the two different MUC1 aptamer‐amphiphiles. As can be seen in Figure 1(b), the MC4N aptamer‐amphiphiles self‐assembled into spherical micelles with 11 ± 2 nm in diameter (*n* = 100), while the MC12N aptamer‐amphiphiles formed micrometer long cylindrical micelles with a diameter of 13 ± 3 nm (*n* = 60) (Figure 1(c)). The packing parameter, that is commonly used to describe the shapes of self‐assembled molecules, is defined as the (cross‐sectional area of the hydrophobic tail)/(equilibrium area per molecule at the aggregate surface). In our case, the cross‐sectional area of the hydrophobic tail‐spacer, regardless of the alkyl spacer length, is defined by the cross‐sectional area of the 1,8‐naphthalimide, while the equilibrium area occupied by each amphiphile at the aggregate surface is influenced by the steric and electrostatic repulsions present between the aptamer headgroups.^43^ The packing parameter would therefore be identical for the MC4N and MC12N aptamer‐amphiphiles. However, it has also been shown theoretically that for the same value of the packing parameter, the sphere‐to‐rod transition parameter increases with increasing tail length, thus making the formation of cylindrical micelles more favorable for MC12N aptamer‐amphiphiles with the longer C_12_ hydrophobic spacer than the MC4N aptamer‐amphiphiles with the shorter C_4_ spacer.^43^

**FIGURE 2:**
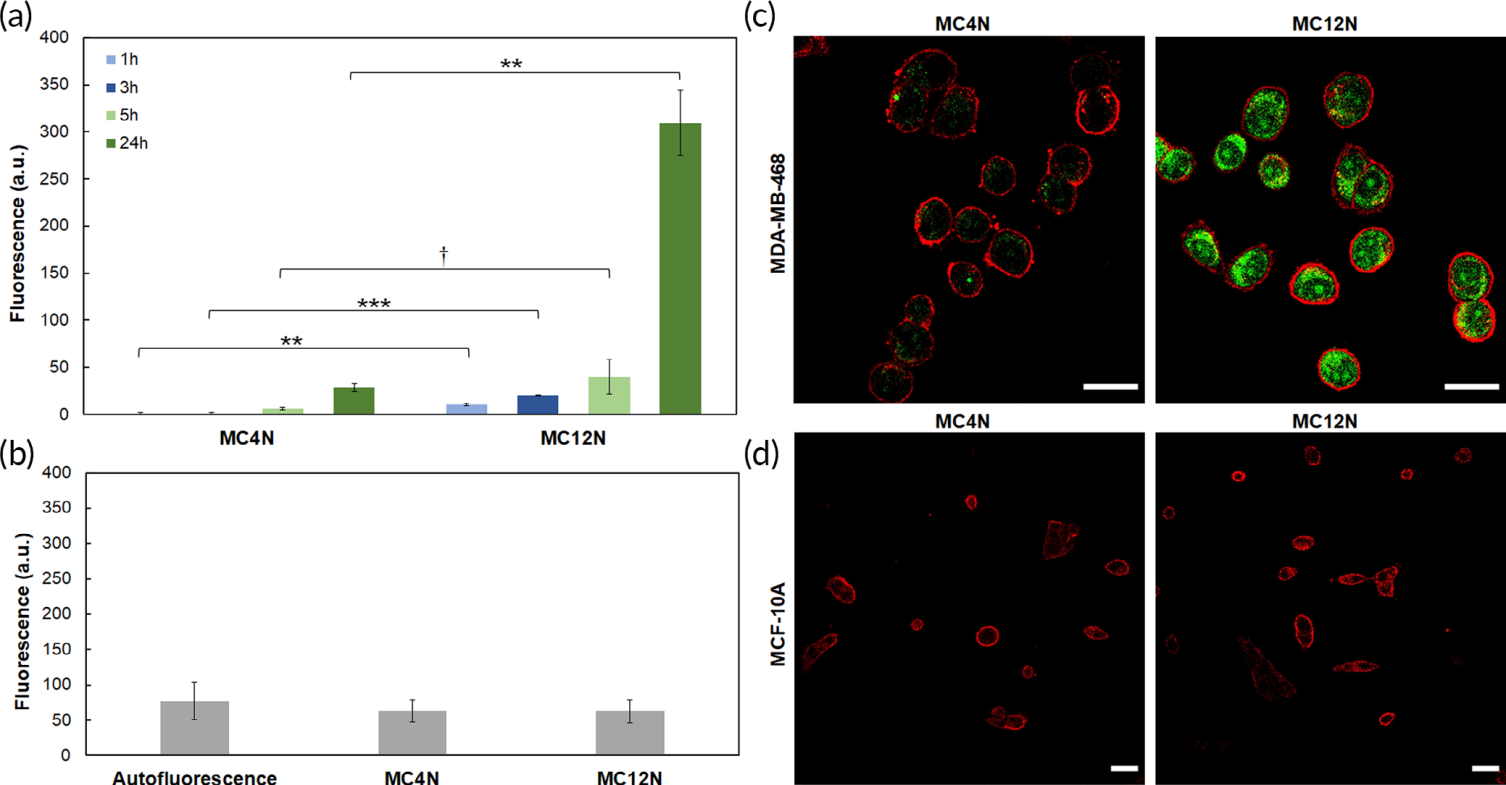
Cell uptake of MC4N spherical micelles and MC12N cylindrical micelles. Flow cytometry results at 37°C for (a) MDA‐MB‐468 cells as a function of time (the cell autofluorescence was subtracted from all samples), and (b) MCF‐10A cells at 24 h (the cell autofluorescence was not subtracted and is shown for comparison). Data are presented as the mean ± SD (*n* = 3). Two‐tailed Student's *t*‐test analysis was used to determine significance, ** *p* < 0.005, *** *p* < 0.00001, † *p* > 0.05. Confocal microscopy images of MC4N spherical micelles and MC12N cylindrical micelles incubated for 24 h at 37°C with (c) MDA‐MB‐468, and (d) MCF‐10A cells. Nanoparticles are shown in green and cell membranes in red. Scale bars are 25 μm

While both self‐assembled nanoparticles should have the inherent targeting ability of the MUC1 aptamer, this distinct difference in structure enables the probing of the impact of the size and shape on their binding and internalization with the TNBC cells. Prior to examining the targeting ability of the aptamer nanoparticles, it was important to first establish whether they were cytotoxic to either a human TNBC cell line, MDA‐MB‐468, or a noncancerous human breast tissue cell line, MCF‐10A. Using an ATP‐based luminescence cell viability assay, it was determined that neither the MC4N spherical micelles nor the MC12N nanofibers were cytotoxic to either cell line after 24 h (Figure S4). The presence of the MUC1 glycoprotein on the cell surface was also evaluated via flow cytometry experiments. Figure S5 demonstrates that the MDA‐MB‐468 overexpressed MUC1, whereas the MCF‐10A showed minimal to no expression. With this confirmation in place, MC4N spherical micelles or MC12N cylindrical micelles were delivered to MDA‐MB‐468 cells and incubated for 1, 3, 5, or 24 h. The results are shown in Figure 2(a). At all times, the MC12N nanofibers showed greater association with TNBC cells than the MC4N spherical micelles (the cell autofluorescence was subtracted from all samples). This suggests that the morphology of the self‐assembled structure formed by the amphiphiles can have an enormous impact on the ability of nanoparticles to associate with cells at the same amphiphile concentration. MC4N spherical micelles or MC12N nanofibers were also delivered to MCF‐10A cells and incubated for 24 h prior to analysis by flow cytometry. Unsurprisingly, considering their lack of MUC1 expression, no association was seen for either the spherical or cylindrical micelles (Figure 2(b)). Cell internalization was further examined via confocal microscopy. MC4N spherical micelles or MC12N cylindrical micelles were delivered to MDA‐MB‐468 cells and incubated for 24 h. Results closely mirror those from flow cytometry. As seen in Figure 2(c), a weak signal representing the fluorescent amphiphiles (green) can be seen on the cytoplasmic side of the cell membrane (red) of MDA‐MB‐468 cells treated with MC4N spherical micelles, while a considerably stronger signal can be seen within the cytoplasm of those cells treated with MC12N nanofibers. These conclusively prove that the MC12N nanofibers internalize in representative TNBC cells to a much greater extent than MC4N spherical micelles. To verify specificity, MCF‐10A cells were also incubated with MC4N or MC12N nanoparticles for 24 h. The results were comparable to the flow cytometry data, with neither group of treated cells showing any nanoparticle internalization (Figure 2(d)).

In addition to conjugating the MUC1 aptamer to the tail via the C_4_ and C_12_ spacers, direct conjugation of the ssDNA to the 1, 8‐naphthalimide was attempted. The high temperatures used in the spacer‐tail reactions would lead to degradation of the aptamer,^44^ so alternative strategies had to be examined. While attempts were made to use other protocols from the literature which utilized lower temperature reactions,^45^, ^46^ the only option which was feasible and produced significant yield was to apply the same reaction scheme used for the spacer‐tail reactions (Scheme 1), but at a lower temperature and for longer time (48 h, 100°C, ssDNA aptamer solubilized in *N*,*N*′‐dimethylformamide via cetyl trimethylammonium bromide). While this produced the best yield of the available options, the yield was still very low (~10%) and the aptamer‐amphiphile proved difficult to separate from the pure ssDNA aptamer. Regardless, the direct conjugate was tested via flow cytometry for cell association after incubation for 24 h with MDA‐MB‐468 cells at same nanoparticle concentration used for Figure 2. However, fluorescence was not above cell autofluorescence. Therefore, given the difficulties in synthesis and failure to show cell association, the aptamer that was directly conjugated to the 1,8‐naphthalimide tail was not pursued further. These results demonstrate the need for the spacer between the naphthalimide tail and the aptamer.

**FIGURE 3:**
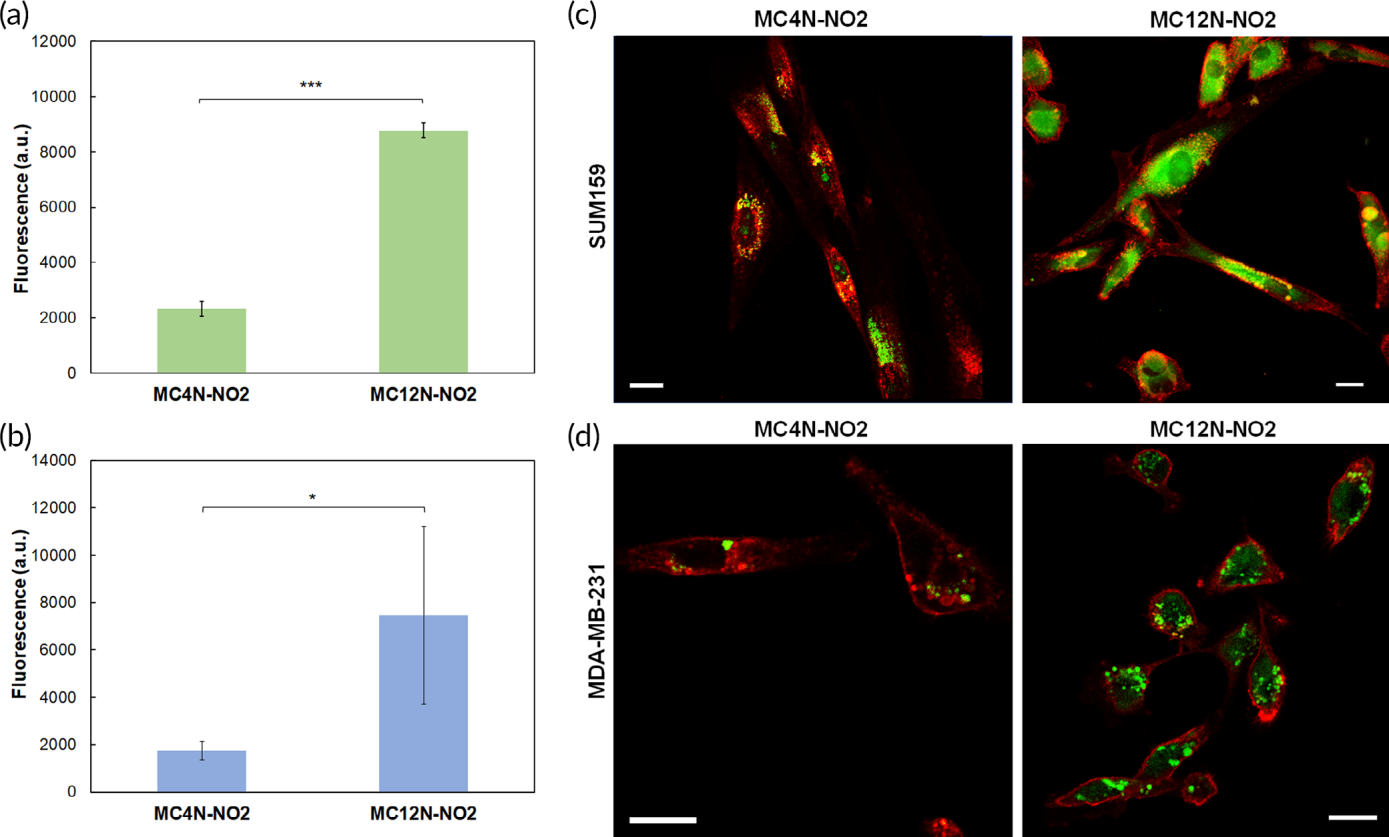
Cell uptake of MC4N‐NO2 spherical micelles and MC12N‐NO2 cylindrical micelles. Flow cytometry results after 24 h at 37°C for (a) SUM159 cells, and (b) MDA‐MB‐231 cells. The cell autofluorescence was subtracted from all samples. Data are presented as the mean ± SD (*n* = 3–5). Two‐tailed Student's *t*‐test analysis was used to determine significance, * *p* < 0.05, *** *p* < 0.00001. Confocal microscopy images of MC4N‐NO2 spherical micelles and MC12N‐NO2 nanofibers incubated for 24 h at 37°C with (c) SUM159, and (d) MDA‐MB‐231 cells. Nanoparticles are shown in green and cell membranes in red. Scale bars are 20 μm

A 4‐substituted naphthalimide tail (4‐nitro‐1,8‐naphthalimide) was used for experiments requiring fluorescence measurements in the rest of paper as fluorescence emission strongly increased (the excitation wavelength was shifted to the visible range), and most importantly, its fluorescent signal did not overlap with fluorescence of blood plasma, an important consideration for the data analysis of the pharmacokinetic study as it enables a lower limit of detection. Fluorescence spectroscopy confirmed that both the MC4N‐NO2 and MC12N‐NO2 aptamer‐amphiphiles exhibited a maximum excitation around 415 nm and emission wavelength of 560 nm (Figure S6). The absorbance and fluorescence of 4‐nitro‐1,8‐naphthalic anhydride is also shown in Figure S6. Comparison of Figures S3(b) and S6(b) show a much higher fluorescence for MC4N‐NO2 and MC12N‐NO2 aptamer‐amphiphiles compared to MC4N and MC12N. To examine if MUC1 aptamer nanoparticles can be used to target other TNBC cells, SUM159 and MDA‐MB‐231 cells were used to evaluate uptake of MC4N‐NO2 and MC12N‐NO2 nanoparticles. First, MUC1 glycoprotein surface expression was evaluated via flow cytometry, and Figure S7 demonstrates that SUM159 and MDA‐MB‐231 overexpress MUC1. Nanoparticle uptake was evaluated via flow cytometry and confocal microscopy, and Figure 3 verifies that the MUC1 aptamer nanoparticles bind and internalize into both SUM159 and MDA‐MB‐231 cells, with the MC12N‐NO2 showing significantly higher cell association and internalization compared to the MC4N‐NO2 nanoparticles.

Our work demonstrates that the overall shape and size of the self‐assembled structures dramatically impacts cell association and internalization behavior in the presence of a targeting aptamer. There is evidence in the literature regarding the effect of physical properties such as size, shape, and stiffness on cell uptake.^47^ For example, gold and some polymeric rod‐like or cylindrical nanoparticles showed reduced cellular uptake compared to spheres, while targeted rod‐like polystyrene nanoparticles coated with antibodies exhibited higher specific cell uptake than their spherical counterparts.^20^, ^32^ Mathematical modeling showed that elongated particles coated with ligands can form more substantial contacts with their targeting receptors, compared to spheres, due to the engagement of multiple ligand‐ receptor interactions.^48^

**FIGURE 4:**
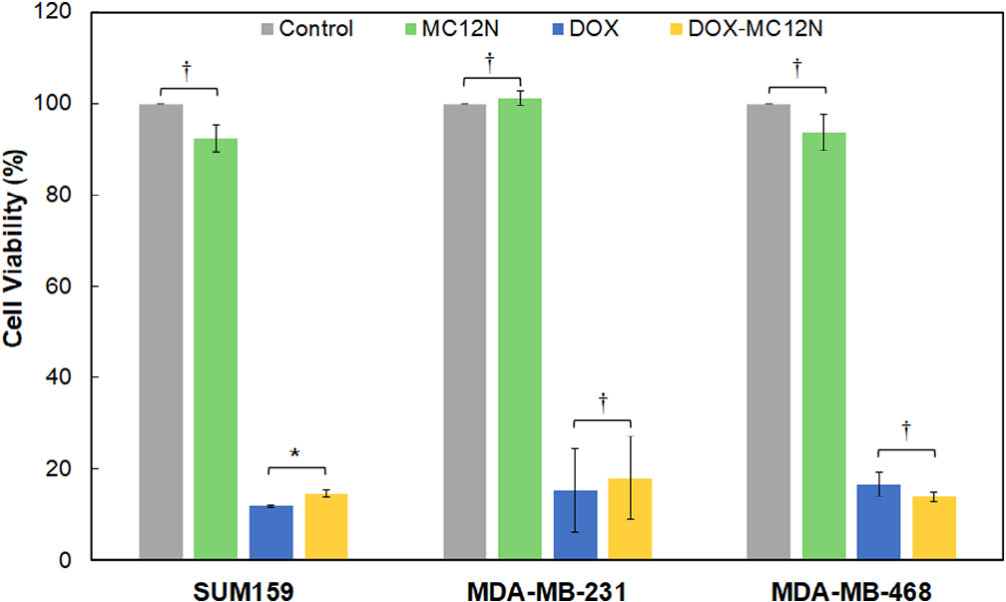
Cell viability of triple negative breast cancer (TNBC) cells after treatment with different samples (14 μM MC12N, 5 μg/ml DOX, or 14 μM MC12N with 5 μg/ml DOX) for 12 h at 37°C. The cells were washed and allowed to incubate for an additional 36 h at 37°C. Data are shown as percentage of untreated cells and presented as mean ± SD (*n* = 3, done in triplicate). Two‐tailed Student's *t*‐test analysis was used to determine significance, * *p* < 0.05, † *p* > 0.05

Overall, the data suggest that the self‐assembled MUC1 aptamer nanofibers internalize specifically into different TNBC cells with great affinity and can be seen without additional staining due to the presence of the naphthalimide tail. Thus, the MC12N aptamer nanofibers were used further to examine the ability of these nanoparticles to deliver a therapeutic load, such as DOX, to the TNBC cells. DOX has been shown to intercalate into the double‐stranded regions of stem‐loops, thus forming physical complexes with aptamers through noncovalent intercalations.^49^ For the formation of the DOX‐MC12N complexes, MC12N aptamer nanofibers were disassembled and allowed to re‐assemble in the presence of DOX, followed by removal of free DOX by dialysis. The MC12N cylindrical micelles were not toxic to the cells on their own, whereas when used to deliver DOX to MDA‐MB‐468, SUM159, and MDA‐MB‐231 cells, they were as cytotoxic as free DOX (Figure 4).

**FIGURE 5:**
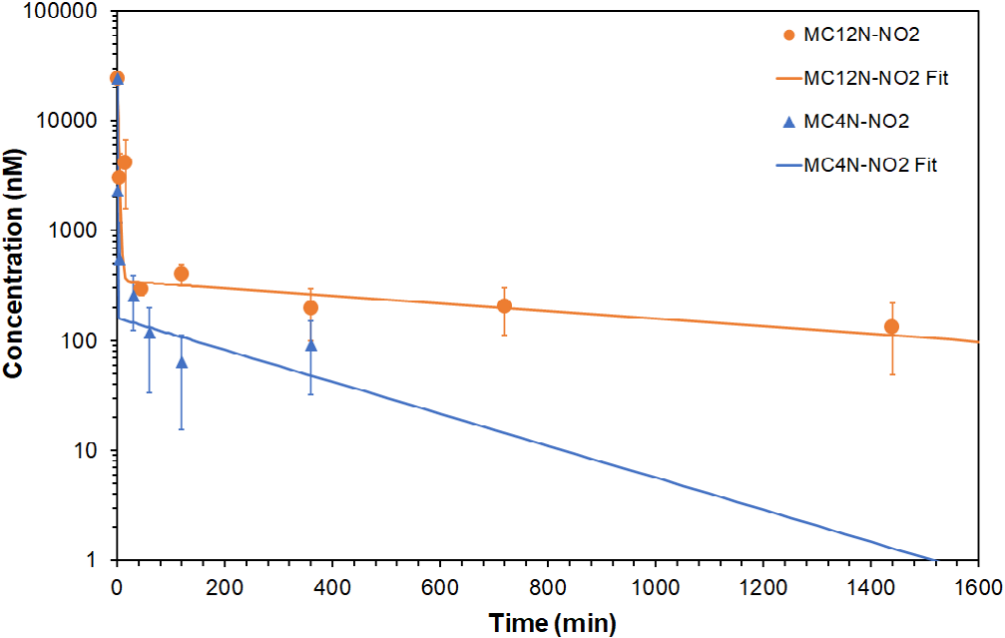
In vivo clearance of MUC1 aptamer nanoparticles. Plasma aptamer‐amphiphile concentration versus time after intravenous injection. Data are presented as mean ± SD (*n* = 3–4). The lines are fits to a two‐compartment model

Finally, to demonstrate the promise of the MUC1 aptamer nanoparticles as delivery vehicles, a pharmacokinetic study was performed in mice. The fluorescence of MC4N and MC12N nanoparticles overlapped with the fluorescence of blood plasma from mice, in contrast to the MC4N‐NO2 and MC12N‐NO2 nanoparticles. Therefore, MC4N‐NO2 and MC12N‐NO2 nanoparticles were administered via the tail vein and their concentration in the blood was measured (Figure 5). A two‐compartment model was used to fit the data (Figure 5), and the calculated pharmacokinetic parameters for both nanoparticles are shown in Table 1. The elimination half‐life of 238.4 ± 94.9 min of the MC4N‐NO2 spherical micelles, without any extra stabilization, compares favorably with spherical nucleic acids (ssDNA chemisorbed on gold nanoparticles) stabilized with ethylene oxide, that have an average elimination half‐life of 400.6 min.^50^ The pharmacokinetic results demonstrate the much longer circulation time and slower plasma elimination rate of the MC12N‐NO2 long nanofibers compared to the MC4N‐NO2 spherical micelles. The elimination half‐life of the aptamer nanofibers (18.1 ± 10.1 h), exceeded the elimination half‐life of the aptamer micelles (4.0 ± 1.6 h), demonstrating a 4.6‐fold increase. Likewise, the area under the curve value and clearance of the MUC1 long nanofibers was 8.2 times higher and 10 times lower, respectively, than those of the spherical micelles. This result is in agreement with work from the literature showing that filaments have a much longer circulation time than spherical nanoparticles.^51^

**TABLE 1:** Pharmacokinetic parameters in mice after intravenous administration of MUC1 aptamer nanoparticles (mean ± SD, *n* = 3–4)

Particle	Half‐life of distribution, αt_1/2_ (min)	Half‐life of elimination, βt_1/2_ (min)	AUC (nM × min)	Clearance, CL (ml/min/kg)
MC4N‐NO2	0.5 ± 0.5	238.4 ± 94.9	74,138 ± 48,616	39.1 ± 28.3
MC12N‐NO2	1.6 ± 0.5	1085.2 ± 605.4	609,573 ± 292,281	3.9 ± 2.2

Abbreviation: AUC, area under the curve.

This work lends further credence to the significant impact size and shape can have on targeted delivery and the growing body of evidence that with the appropriate morphology, one can achieve increased specific targeting and internalization into cancer cells, as well as longer circulation times. Thus, the MUC1 aptamer nanoparticles designed in this study present a promising platform for targeted delivery.

## 3 CONCLUSIONS

Given the difficulty of treating TNBC, the development of a targeted nanoparticle delivery system would be highly impactful. Such a system would enable enhanced efficacy and greatly reduced off‐target effects for a variety of treatment options including gene therapy and traditional chemotherapeutics. In this work, a MUC1 aptamer‐amphiphile was designed which provides targeting, an entry mechanism into the MUC1‐expressing TNBC cells, and visualization capabilities due to its naphthalimide tail. The use of a MUC1 aptamer could also be used with a wide variety of cancers, as nearly all carcinomas overexpress MUC1. Two different amphiphile constructs were generated to examine the impact of the spacer length on self‐assembly and cell association. Cryo‐TEM images demonstrated that the short C_4_ alkyl spacer led to the production of spherical micelles, while the C_12_ alkyl spacer led to micrometer long cylindrical micelles. The targeting was highly effective, with neither construct showing association with the MCF‐10A cells, while both vehicles showing significant internalization into the TNBC cells. That said, the cell internalization of the MC12N nanofibers was greatly enhanced over that of the MC4N spherical micelles, suggesting that the shape of the aptamer nanoparticle plays an enormous role in its ability to interact and internalize with the cancer cell. In addition, it was shown that the MUC1 aptamer nanofibers could be used effectively for the delivery of a chemotherapeutic, such as DOX, and had a more favorable pharmacokinetic profile than the MUC1 spherical micelles, thus making them a promising targeted drug delivery system.

## 4 CONFLICT OF INTEREST

The authors declare no conflict of interest.

## Supporting information


**Appendix S1:** Supporting information
