# Supplementary material for: Effect of an alkyl spacer on the morphology and internalization of MUC1 aptamer‐naphthalimide amphiphiles for targeting and imaging triple negative breast cancer cells
Source: Bioeng Transl Med. 2020 Nov 10;6(1):e10194. doi: 10.1002/btm2.10194 (PMC7823120; doi:10.1002/btm2.10194)
Supplement: Supplementary file 1 — Appendix S1: Supporting information [file BTM2-6-e10194-s001.pdf]

Supplementary Information

Effect of an alkyl spacer on the morphology and internalization of MUC1 aptamer-naphthalimide amphiphiles for targeting and imaging triple negative breast cancer cells

Huihui Kuang[1†], Zachary Schneiderman[1,2†], Ahmed M. Shabana[1,3], Gabriella C. Russo[1,2], Jun Guo[1], Denis Wirtz[1,2], Efrosini Kokkoli[1,2*]

[1] Institute for NanoBioTechnology, Johns Hopkins University, Baltimore, MD 21218, USA

[2] Department of Chemical and Biomolecular Engineering, Johns Hopkins University, Baltimore, MD 21218, USA

[3] Department of Pharmaceutics and Industrial Pharmacy, Faculty of Pharmacy, Cairo University, Cairo 11562, Egypt

[†] H.K. and Z.S. contributed equally to this work

[*] Corresponding author: kokkoli@jhu.edu

MATERIALS AND METHODS

Materials. *N,N'*-Dimethylformamide (DMF), ethyl acetate, methanol, dichloromethane (DCM) and triethylamine (TEA) were purchased from Fischer Chemical (Hanover Park, IL). MUC1 aptamer (5'-NH_2-C_6AAGGGATGACAGGATACGCCAAGCT-3') was purchased from Integrated DNA Technologies (Coralville, IA), CellTiter-Glo 2.0 Cell Viability Assay from Promega (Madison, WI), cetyl trimethylammonium bromide (CTAB) from Acros Organics (Morris Plains, NJ), and hexafluroisopropanol (HFIP) from Oakwood Products Inc. (West Columbia, SC). Doxorubicin-hydrogen chloride (DOX) was purchased from Alfa Aesar (Haverhill, MA). Lacey Formvar/carbon 200 mesh copper grids were purchased from Ted Pella Inc. (Redding, CA). All other chemicals and materials were purchased from Sigma-Aldrich (St Louis, MO).

Synthesis of naphthalimide tails. Carboxyl-modified naphthalimides with different spacer lengths (C_4 or C_{12}) were synthesized as depicted in **Scheme 1**.[1] A mixture of 1,8-naphthalic

anhydride (**1a**, 1.98 g, 10 mmol) or 4-nitro-1,8-naphthalic anhydride (**1b**, 2.43 g, 10 mmol) and 4-aminobutanoic acid (**2a**, 1.03 g, 10 mmol) or 12-aminododecanoic acid (**2b**, 2.15g, 10 mmol) was heated at reflux in 100 mL DMF overnight. Upon addition of the hot reaction mixture to ice and cold water, the product (**3**) precipitated. For the reactions involving the non-substituted tail, the resulting precipitate was filtered, washed with diethyl ether, and air-dried. For those involving the substituted tail, the resulting precipitate was isolated via centrifugation, washed with diethyl ether, and air-dried. NHS activated naphthalimide tails were synthesized according to literature.[2] N-hydroxysuccinimide (NHS, 1.5x molar excess) was added to a solution of **3** in DCM at room temperature. After cooling to 0 ºC, *N,N'*-dicyclohexylcarbodiimide (DCC, 2x molar excess) was added. The solution was stirred for 1 h at 0 ºC and then overnight at room temperature. The precipitated dicyclohexyl urea (DCU) was filtered off, and solvent was removed in vacuum. Product **4** was recrystallized from ethyl acetate.

^{1}H NMR spectra were recorded on a Bruker Avance 300 MHz spectrometer (NMR Core Facility at the Johns Hopkins University, Department of Chemistry) with deuterated chloroform ($CDCl_3$) as a solvent at room temperature. Proton chemical shifts were referenced to the deuterated chloroform solvent peak (7.26 ppm, in $CDCl_3$).

Products **3a**,**b** and **4a**,**b**,**c**,**d** were characterized with ^{1}H NMR (300 MHz, $CDCl_3$) δ (ppm):
(**3a**): 8.62 (dd, 2H, *napht*, *J* = 7.3, 1.1 Hz), 8.23 (dd, 2H, *napht*, *J* = 8.3, 1.2 Hz), 7.76 (t, 2H, *napht*, *J* = 7.8 Hz), 4.27 (t, 2H, N-CH_2, *J* = 7.0 Hz), 2.47 (t, 2H, CH_2-COOH, *J* = 7.4 Hz), 2.09 (quint, 2H, CH_2-CH_2-CH_2, *J* = 7.3 Hz).
(**3b**): 8.62 (dd, 2H, *napht*, *J* = 7.3, 1.2 Hz), 8.22 (dd, 2H, *napht*, *J* = 8.3, 1.1 Hz), 7.78 (dd, 2H, *napht*, *J* = 8.3, 7.3 Hz), 4.17 (t, 2H, N-CH_2, *J* = 7.6 Hz), 2.34 (t, 2H, CH_2-COOH, *J* = 7.5 Hz), 1.72 (quint, 2H, N-CH_2-CH_2, *J* = 7.9 Hz), 1.61 (quint, 2H, CH_2-CH_2-COOH, *J* = 7.3 Hz), 1.28 (m, 14H, N-CH_2-CH_2-(CH_2)$_7$-CH_2-CH_2-COOH).
(**4a**): 8.63 (dd, 2H, *napht*, *J* = 7.2, 1.1 Hz), 8.24 (dd, 2H, *napht*, *J* = 8.3, 0.9 Hz), 7.77 (dd, 2H, *napht*, *J* = 8.3, 7.3 Hz), 4.33 (t, 2H, N-CH_2, *J* = 7.0 Hz), 2.87-2.73 (m, 6H, NHS, -CH_2-COO), 2.22 (quint, 2H, CH_2-CH_2-CH_2, *J* = 7.3 Hz).
(**4b**): 8.62 (dd, 2H, *napht*, *J* = 7.3, 1.2 Hz), 8.22 (dd, 2H, *napht*, *J* = 8.3, 1.2 Hz), 7.78 (dd, 2H, *napht*, *J* = 8.3, 7.3 Hz), 4.17 (t, 2H, N-CH_2, *J* = 7.5 Hz), 2.83 (s, 4H, NHS, *J* = 2.8 Hz), 2.59 (t, 2H, CH_2-COO, *J* = 7.5 Hz), 1.73 (quint, 4H, N-CH_2-CH_2,CH_2-CH_2-COO, *J* = 7.4 Hz), 1.27 (m, 14H, N-CH_2-CH_2-(CH_2)$_7$-CH_2-CH_2-COO).
(**4c**): 8.85 (dd, 1H, *napht*, *J* = 8.8, 1.0 Hz), 8.76 (dd, 1H, *napht*, *J* = 7.3, 1.0 Hz), 8.70 (d, 1H, *napht*, *J* = 7.8 Hz), 8.41 (d, 1H, *napht*, *J* = 7.8 Hz), 7.99 (dd, 1H, *napht*, *J* = 8.5, 7.3 Hz), 4.33 (t, 2H, N-CH_2, *J* = 7.1 Hz), 2.81-2.77 (m, 6H, NHS, CH_2-COO), 2.22 (quint, 2H, CH_2-CH_2-CH_2, *J* = 7.3 Hz)
(**4d**): 8.84 (dd, 1H, *napht*, *J* = 8.7, 1.0 Hz), 8.74 (dd, 1H, *napht*, J = 7.3, 1.0Hz), 8.69 (d, 1H, *napht*, *J* = 8.0 Hz, 8.41 (d, 1H, *napht*, *J* = 8.0 Hz), 7.99 (dd, 1H, *napht*, *J* = 8.7, 7.3 Hz), 4.18 (t, 2H, N-CH_2, *J* = 7.6 Hz), 2.83 (s, 4H, *NHS*), 2.59 (t, 2H, CH_2-COO, *J* = 7.5 Hz), 1.73 (quint, 4H, N-CH_2-CH_2,CH_2-CH_2-COO, *J* = 7.5 Hz), 1.28 (m, 14H, N-CH_2-CH_2-(CH_2)$_7$-CH_2-CH_2-COO).

Synthesis and characterization of MUC1 aptamer-amphiphiles. MUC1 aptamer-amphiphiles were synthesized by solution coupling according to previous work in our group (**Scheme 1**).[3] 1.5x molar excess CTAB dissolved in water was added to the ssDNA aptamer. Here the NH_4^+

ammonium moiety of CTAB is electrostatically attracted to the PO_4^- of the ssDNA backbone, forming a sheath of hydrocarbon chains that surround the hydrophilic ssDNA molecule, which renders the ssDNA soluble in DMF. A 10x molar excess of NHS-activated tails were added to the CTAB-ssDNA complexes dissolved in DMF and the reaction was stirred at 50 °C for 24 h. After 24 h, the DMF was removed by evaporation and the aptamer-amphiphiles and any unreacted ssDNA were purified by lithium perchlorate (2.5% in acetone) precipitation to remove unreacted tails and CTAB. Unreacted ssDNA was separated from the ssDNA-amphiphile using reverse-phase high performance liquid chromatography (RP-HPLC) after they were dissolved in water and filtered (Zorbax C3 300 Å SB column, 5-95% B over 30 min, buffer A: H_2O, 100 mM HFIP, 14.4 mM TEA, buffer B: Methanol, 100 mM HFIP, 14.4 mM TEA). To confirm the success of the synthesis, the molecular weights of the aptamer-naphthalimide amphiphiles were verified via liquid chromatography-mass spectroscopy (LC-MS) acquired with a Zorbax C8 300 Å SB column (50-80% B over 15 min, buffer A: H_2O+15 mM ammonium acetate, buffer B: acetonitrile) and an Agilent MSD ion trap.

Fluorescence of MUC1 aptamer-amphiphiles and naphthalic anhydrides. 100 μM of all aptamer-amphiphiles in Milli-Q water were prepared for the readings. The absorbance and fluorescence of the naphthalic anhydrides were also investigated. The 1,8-naphthalic anhydride was dissolved in pure MeOH at 0.8 mg/mL and diluted with Milli-Q water to either 100 μM for fluorescence readings, or 800 μM for absorbance readings (higher concentration required to achieve sufficient signal). The 4-nitro-1,8-naphthalic anhydride was dissolved in pure MeOH at 0.6 mg/mL and diluted with Milli-Q water to 100 μM for fluorescence readings, and 300 μM for absorbance. 2 μL of the amphiphiles or naphthalic anhydrides were placed in the Take3 plate of a BioTek Synergy H1 microplate reader (Winooski, VT), and absorption profiles and fluorescence emission spectra were collected. For the 1,8-naphthalic anhydride and MC4N and MC12N aptamer-amphiphiles, an excitation wavelength of 300 nm was used rather than the max absorbance of 345 nm, to avoid signal coming from the excitation source. For the 4-nitro-1,8-naphtahlic anhydride and MC4N-NO2 and MC12N-NO2 aptamer-amphiphiles, an excitation wavelength of 415 nm was used. While the max absorbance for 4-nitro-1,8-naphtahlic anhydride was 345 nm, excitation at this wavelength generated negligible fluorescence, thus 415 nm excitation was used.

Circular dichroism (CD). 500 μM of MC4N and MC12N aptamer-amphiphiles or free aptamer solutions were diluted to 50 μM with Milli-Q water and transferred to a 0.1 cm path length cuvette. CD spectra were collected using an AVIV 420 CD spectrometer (Center for Molecular Biophysics at the Johns Hopkins University) using a read speed of 50 nm/min in 1 nm steps. Three accumulations per sample solution were recorded with the background spectrum from the water automatically subtracted. The accumulations were averaged and the raw ellipticity values were converted to molar ellipticity. CD data were smoothed with the Sovitsky-Golay function on Matlab (order: 3 field: 11).

Cryogenic transmission electron microscopy (cryo-TEM). 5 μL of MC4N and MC12N aptamer-amphiphile solutions (500 μM in Milli-Q water) were deposited onto lacey formvar/carbon copper grids that had been treated with glow discharge for 60 s and vitrified in liquid ethane by Vitrobot (Vitrobot parameters: 5 s blot time, 0 offset, 3 s wait time, 3 s relax time, 95% humidity). After vitrification, the grids were kept under liquid nitrogen and were transferred

to a FEI Tecnai 12 TWIN transmission electron microscope operated at an acceleration voltage of 120 kV (Integrated Imaging Center at the Johns Hopkins University, Institute for NanoBioTechnology). Images were captured using an Eagle 2k CCD camera.

Cell Culture. MCF-10A cells were cultured at 37 °C and 5% CO_2 using Mammary Epithelial Cell Basal Medium (MEBM, Lonza, Catalog No: CC-3151) with additives (MEGM Kit, Lonza, Catalog No: CC3150) and 100 ng/mL cholera toxin. MDA-MB-468, MDA-MB-231 and SUM159 cells were cultured at 37 °C and 5% CO_2 using Dulbecco's Modified Eagle Medium (DMEM) (Thermo Fisher Scientific, Rockford, IL) supplemented with 10% (v/v) fetal bovine serum (FBS) (Thermo Fisher Scientific, Rockford, IL), 100 units/mL penicillin and 0.1 mg/mL streptomycin. Cells were passaged when they reached 80% confluence by treatment with TrypLE Express cell dissociation agent (Thermo Fisher Scientific, Rockford, IL) or 0.05% trypsin with 0.53 mM EDTA.

Cytotoxicity of MUC1 aptamer-amphiphiles. MCF-10A and MDA-MB-468 cells were used to evaluate the biocompatibility of MC4N and MC12N aptamer-amphiphiles using the CellTiter-Glo 2.0 assay (Promega Corporation, Madison, WI). The cells were seeded in black 96-well tissue culture treated plates at a density of 5,000 cells/well in 100 μL of media and allowed to adhere for 24 h at 37 ºC. The next day, media was removed, 95 μL of new media and 5 μL of each aptamer-amphiphile (10 nmol in 1X PBS) were added to the wells at a final amphiphile concentration of 100 μM. After incubation with cells for 24 h, the cells were taken out of the 37 °C incubator and allowed to equilibrate to room temperature, while the CellTiter-Glo 2.0 solution was placed in a room temperature water bath. 100 μL of the CellTiter-Glo 2.0 solution was added to each well and the entire plate was placed on an orbital shaker for 2 min and then allowed to rest for 10 min under aluminum foil. The luminescence signal of each well was measured on the BioTek Synergy H1 microplate reader (Winooski, VT), and the luminescence of each group was normalized to the luminescence of the untreated cells.

MUC1 glycoprotein expression via flow cytometry. MUC1 glycoprotein expression on MCF-10A, MDA-MB-468, MDA-MB-231 and SUM159 cells was assessed by flow cytometry. $2x10^5$ cells were each suspended in 200 μL of binding buffer consisting of 1X PBS supplemented with 1% (w/v) bovine serum albumin (PBS/BSA) and 5 μg/mL of anti-MUC1 antibody (MAB6298, R&D Systems, Minneapolis, MN) or a mouse IgG2B isotype control (MAB004, R&D Systems, Minneapolis, MN) were added. After incubating for 30 min at 4 °C, the cells were centrifuged, washed with PBS/BSA and suspended in 200 μL of PBS/BSA with 5 μg/mL of a fluorescently labeled secondary antibody (anti-mouse IgG2B PE-conjugated antibody, F0132, R&D Systems, Minneapolis, MN). The cells were incubated with the secondary antibody for 30 min at 4 °C, washed twice with PBS/BSA, and assessed for MUC1 expression by flow cytometry (LSR II, Ross Flow Cytometry Core Facility at the Johns Hopkins University, Department of Medicine).

Nanoparticle cell association via flow cytometry. MC4N and MC12N aptamer-amphiphiles were prepared at 500 μM in 1X PBS. $2x10^5$ MCF-10A cells or MDA-MB-468 cells were added to a 24-well plate and allowed to attach and proliferate for 24 h. After 24 h, 180 μL of fresh media and 20 μL of MC4N or MC12N aptamer-amphiphiles (10 nmol in 1X PBS) were added at a final amphiphile concentration of 50 μM. After an additional 24 h at 37 °C the amphiphile solution was removed, the cells were detached from the plate, washed with PBS, and analyzed via flow cytometry (LSR II, Ross Flow Cytometry Core Facility at the Johns Hopkins University,

Department of Medicine). This experiment was repeated with MC4N-NO2 and MC12N-NO2 aptamer-amphiphiles and MDA-MB-231 and SUM159 cells. For MDA-MB-468 cells, shorter incubation times (1, 3 and 5 h) with the MC4N and MC12N aptamer-amphiphiles were also evaluated.

Nanoparticle cell internalization via confocal microscopy. MC4N and MC12N aptamer-amphiphiles were prepared at 500 μM in 1X PBS. $2x10^4$ MCF-10A cells or MDA-MB-468 cells were deposited onto glass coverslips contained within wells of a 24-well plate and allowed to attach and proliferate for 24 h. After 24 h, 180 μL fresh media and 20 μL of aptamer-naphthalimide amphiphiles (10 nmol in 1X PBS) were added at a final amphiphile concentration of 50 μM. 24 h after the amphiphiles were added at 37 °C, cells were fixed with 4% paraformaldehyde and their membranes was stained for 15 min with Wheat Germ Agglutinin AlexaFluor594 (Thermo Fisher Scientific, Rockford, IL) at 5.0 μg/mL for the membrane. Cells were mounted onto glass slides using Prolong Gold and imaged with an LSM 700 confocal microscope (Integrated Imaging Center at the Johns Hopkins University, Institute for NanoBioTechnology). This experiment was repeated with MC4N-NO2 and MC12N-NO2 aptamer-amphiphiles and MDA-MB-231 and SUM159 cells.

Doxorubicin (DOX) study. DOX was dissolved in Milli-Q water at 1.5 mM and combined on an equimolar basis with 50 nmol of MC12N amphiphiles in Milli-Q water. DMSO was added to the solution to induce disassembly of the aptamer-amphiphile cylindrical micelles, until the final DMSO concentration was 50% (v/v). The solution was stirred for 2 h. Over 4 additional h, Milli-Q water was slowly added, so that the cylindrical micelles assemble again in the presence of DOX, until the final concentration was 90% water, 10% DMSO (v/v) at the end of the 4 h. The mixture was dialyzed overnight against 1 L Milli-Q water using a 3,500 MWCO Pur-A-Lyzer Maxi Dialysis Kit (Sigma, St. Louis, MO) to remove any free DOX and remaining DMSO. UV-Vis absorbance measurements were used to evaluate concentrations. Since both ssDNA and DOX absorb light at the wavelength of max absorbance for ssDNA, 260 nm, a second wavelength had to be utilized to parse out the individual contributions of each to the measured signal. Therefore, the absorbance of DOX-MC12N was measured at both 260 nm and 488 nm, the maximum absorbance wavelengths for ssDNA and DOX respectively. The extinction coefficient of the MUC1 aptamer at 260 nm was provided by IDT as 256,400 $cm^{-1}M^{-1}$ and can be assumed to remain the same after the attachment of the hydrophobic tail. The extinction coefficient of the MUC1 aptamer at 488 nm was calculated by measuring the absorbance of a known amount of aptamer at both 260 nm and 488 nm, thus providing an extinction coefficient at 488 nm of 25 $cm^{-1}M^{-1}$. Similarly, the extinction coefficients for DOX at 260 nm and 488 nm were calculated to be 14,715 $cm^{-1}M^{-1}$ and 10,200 $cm^{-1}M^{-1}$, respectively. These extinction coefficients were then used with the absorbance measurements at 260 nm and 488 nm in order to solve a system of equations for the concentrations of both DOX and MC12N in solution. It was assumed that the absorbance of the MC12N and DOX were additive with no interacting terms. The effect of free DOX (5 μg/mL in Milli-Q water), MC12N nanoparticles (14 μM of MC12N amphiphiles in Milli-Q water), and DOX-MC12N nanoparticles (5 μg/mL DOX and 14 μM MC12N amphiphiles in Milli-Q water) on viability of three TNBC cell lines was assessed using the CellTiter-Glo 2.0 assay. 10,000 cells of MDA-MB-468, MDA-MB-231, or SUM159 were deposited into white 96-well tissue culture treated plates with 100 μL of media and allowed to adhere for ~24 h at 37 °C. The next day, the media was removed, 95 μL of fresh media was added and 5 μL of each test sample dissolved in Milli-Q water was added to each well. Untreated cells were used as a control. The samples were

incubated with cells for 12 h at 37 °C. This was followed by a single wash with 100 μL 1X PBS, replenishment with 100 μL of media, and additional incubation at 37 °C for 36 h. The cells were then removed from the 37 °C incubator and allowed to equilibrate to room temperature for 30 min, while the CellTiter-Glo 2.0 solution was placed in a room temperature water bath to thaw. 100 μL of the CellTiter-Glo 2.0 solution was added to each well of cells simultaneously and the entire plate was placed on an orbital shaker for 2 min and then allowed to rest for 10 min under aluminum foil. The luminescence signal of each well was measured on the BioTek Synergy H1 microplate reader and the luminescence of each treatment group was normalized to the luminescence of the untreated cells for that cell line in order to assess cell viability of each treatment group.

Pharmacokinetic study in mice. Studies using 7 to 8-week-old female NSG (NOD SCID Gamma) mice (Jackson Laboratory, Bar Harbor, ME) were carried out according to protocols approved by the Johns Hopkins University Animal Care and Use Committee in accordance with the NIH Guide for the Care and Use of Laboratory Animals. All mice were housed at a temperature of 25 °C under a 12 h dark/light cycle. Prior to injection, heat lamp was used to dilate the tail veins. At t =0, 100 μL of 400 μM in 1X PBS of MC4N-NO2 or MC12N-NO2 aptamer-amphiphiles were injected into the left lateral tail vein. For both groups, the first two blood collections were taken from the submandibular vein. Subsequent blood collections were taken from the right lateral tail vein. For the MC4N-NO2 group, blood samples were collected at 1, 5, 30, 60, 120, and 360 min, and for the MC12N-NO2 group, at 5, 15, 45, 120, 360, 720, 1440 min (n = 3-4 for each time point). Approximately 30 μL of blood was collected at each time point into heparin-coated tubes (Thermo Fisher Scientific, Rockford, IL). Whole blood was immediately centrifuged for 5 min at maximum speed (13,000 rpm). Plasma was then removed and placed into fresh heparin-coated tubes and stored on ice before being transferred to -80 °C until analysis. Samples were thawed at ambient temperature, briefly and gently centrifuged (to remove plasma from the tube walls), 10 μL of each sample was combined with 90 μL of Milli-Q water, and fluorescence of each sample was read (excitation/emission at 415/560 nm) on a BioTek Synergy H1 microplate reader. Fluorescence readings were correlated to a standard curve to determine the amount of amphiphiles (in nM) in the plasma at each time point. For the preparation of the standard curve, serial dilutions of MC4N-NO2 and MC12N-NO2 in freshly harvested mouse blood (from undosed mice) were performed. Both one- and two-compartment models were used to fit the data, and the two-compartment model (characterized by a biexponential decrease in particle concentration over time), was found to best fit the data in both nanoparticles. An initial data point at t = 0 was included representing 100% of the injected dose in the blood, with the total blood volume taken to be 8% of the mouse weight. Each elimination curve was analyzed individually for half-life of distribution ($\alpha t_{1/2}$), half-life of elimination ($\beta t_{1/2}$), area under the curve (AUC), and clearance (CL).

REFERENCES

1. Reger DL, Debreczeni A, Reinecke B, et al. Highly Organized Structures and Unusual Magnetic Properties of Paddlewheel Copper(II) Carboxylate Dimers Containing the pi-pi Stacking, 1,8-Naphthalimide Synthon. *Inorg Chem.* 2009;48(18):8911-8924.
2. Kuang H, Gartner III TE, Dorneles de Mello M, et al. ssDNA-amphiphile architecture used to control dimensions of DNA nanotubes. *Nanoscale.* 2019;11(42):19850-19861.
3. Pearce TR, Waybrant B, Kokkoli E. The role of spacers on the self-assembly of DNA aptamer-amphiphiles into micelles and nanotapes. *Chem Commun.* 2014;50(2):210-212.

FIGURES

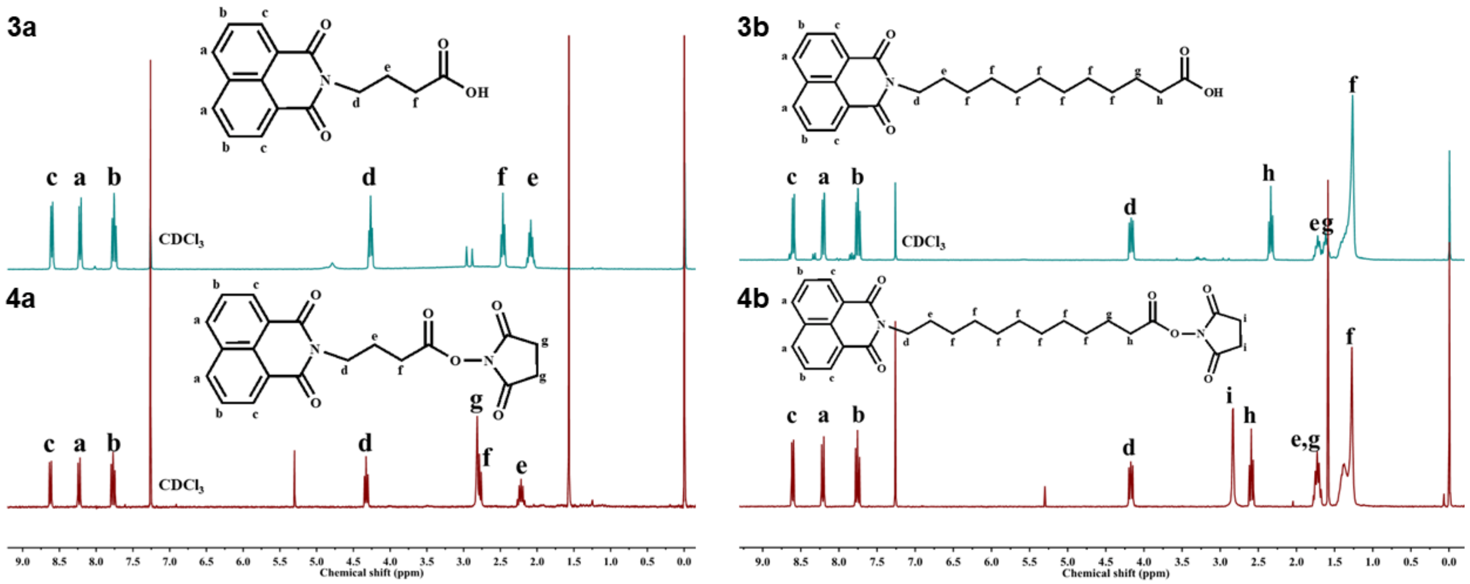

FIGURE S1 ^{1}H NMR of products **3a**,**b** and **4a**,**b** from Scheme 1 (300 MHz, CDCl$_3$)

4c

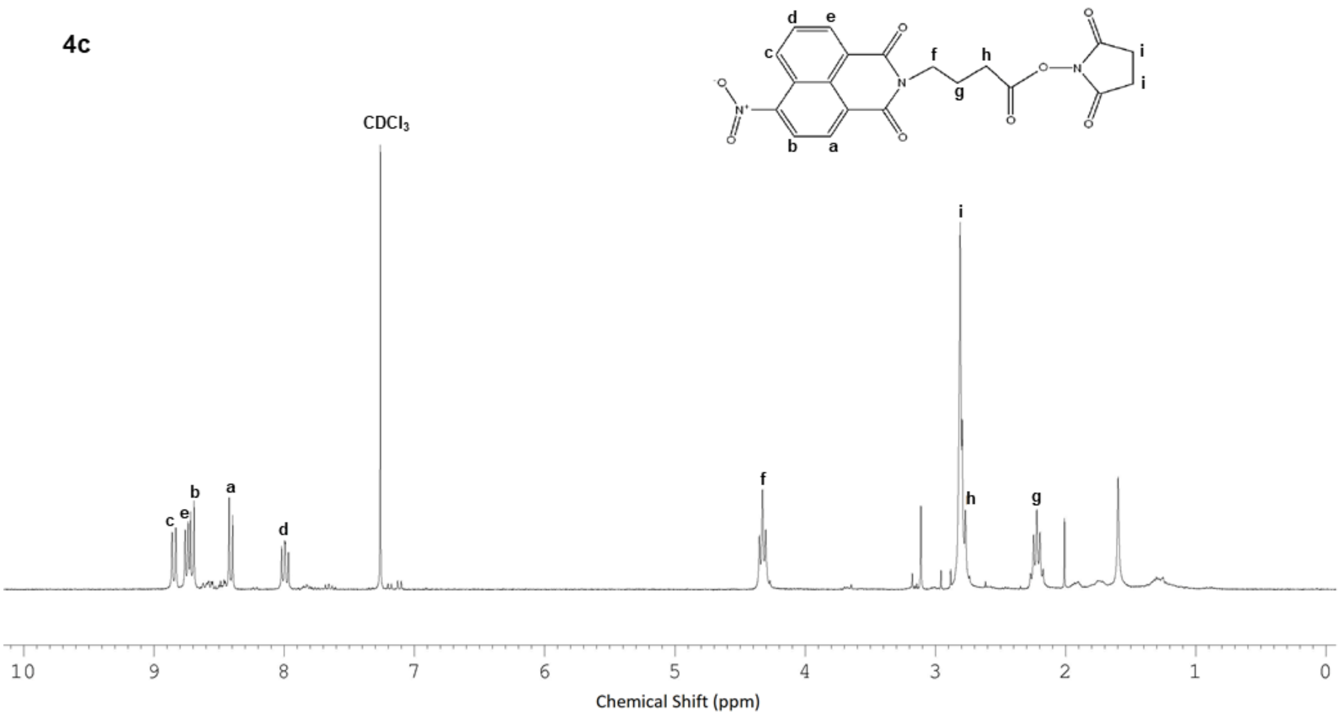

4d

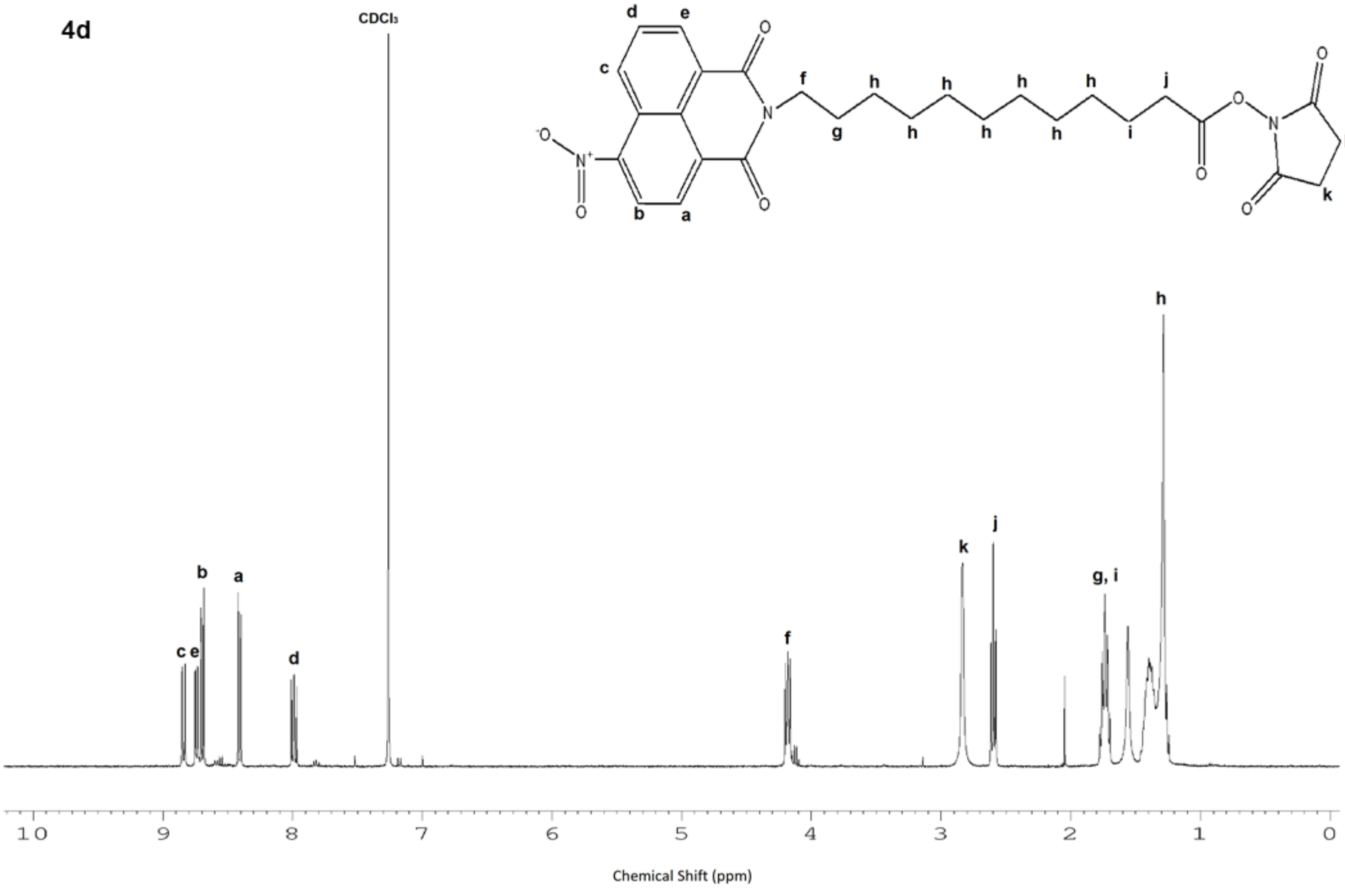

FIGURE S2 ^{1}H NMR of products **4c**,**d** from Scheme 1 (300 MHz, $CDCl_3$)

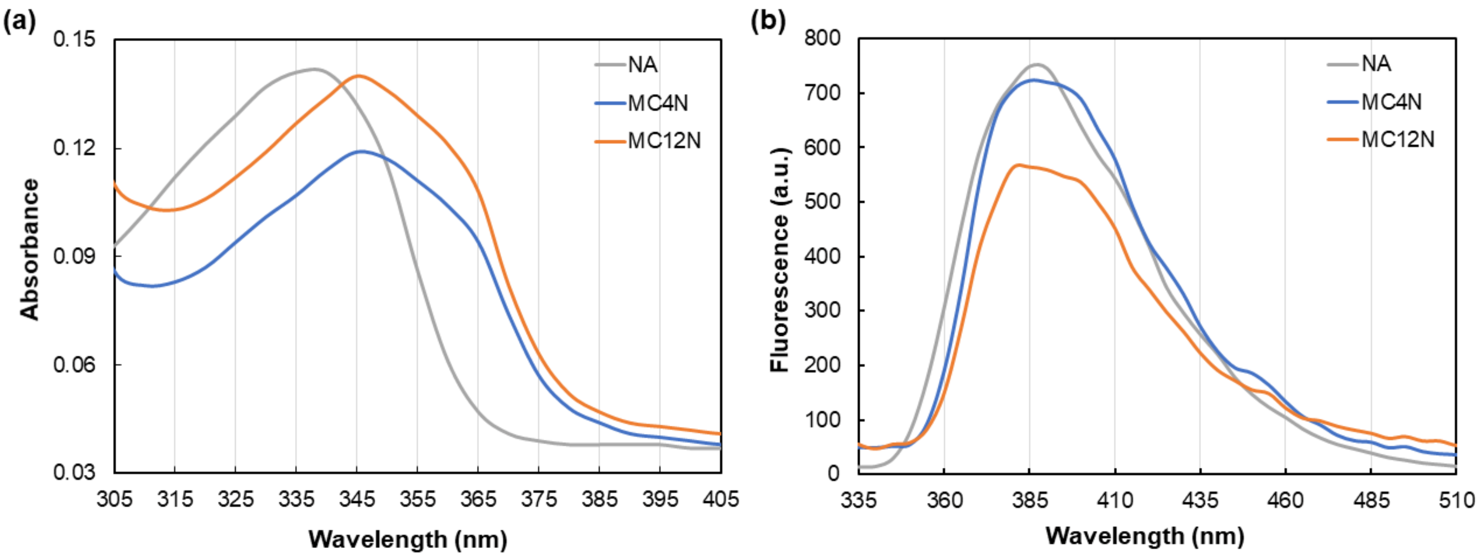

FIGURE S3 (a) Absorption and (b) emission spectra of 1,8-naphthalic anhydride (NA) and MC4N and MC12N aptamer-amphiphiles

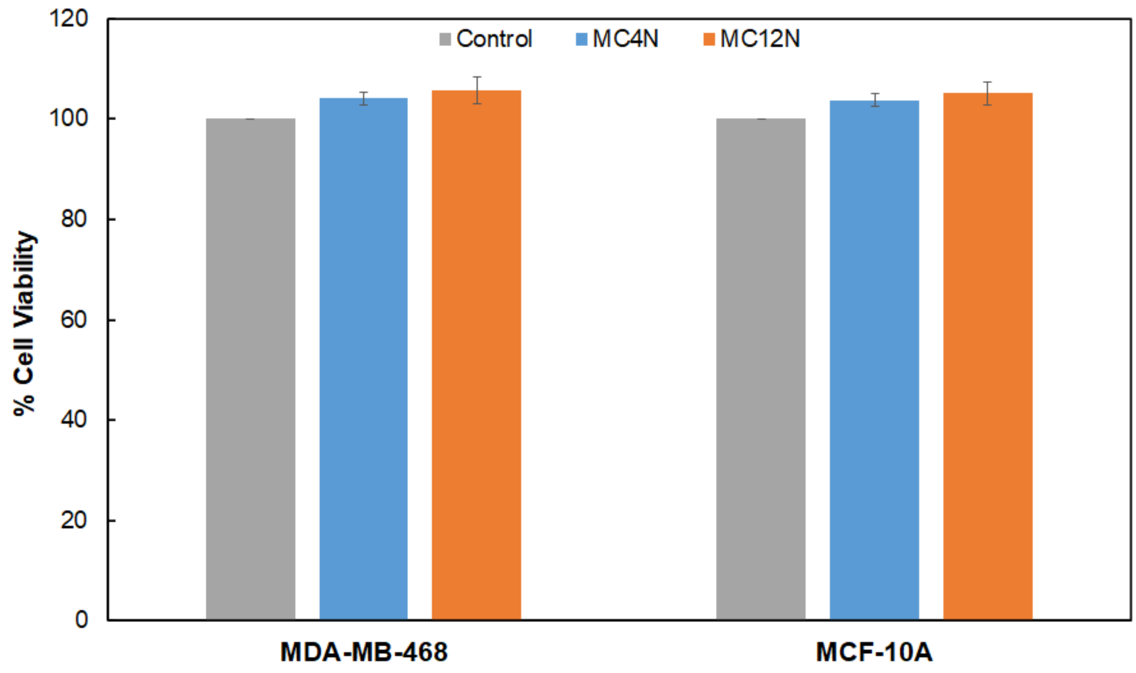

FIGURE S4 Cytotoxicity of MC4N and MC12N nanoparticles to MDA-MB-468 TNBC cells and MCF-10A non-cancerous breast cells after 24 h at 37 °C. Percent cell viability was determined through comparison to PBS treated control cells using an ATP-based luminescence assay. Data are presented as the mean ± SD (n = 3)

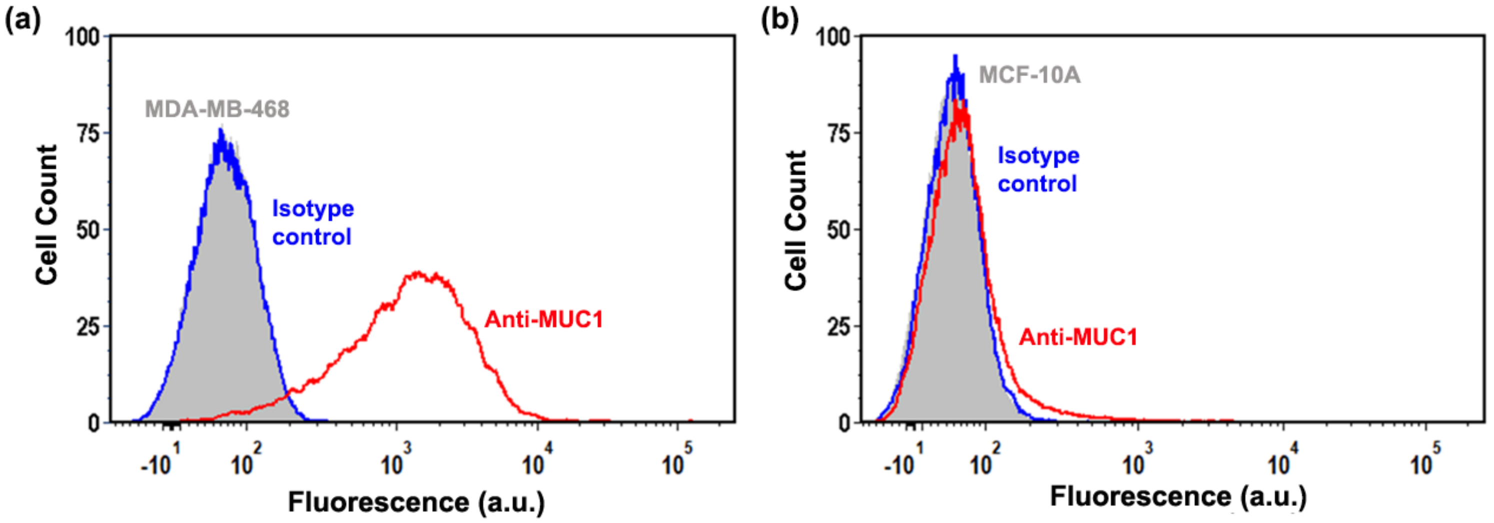

FIGURE S5 MUC1 expression on (a) MDA-MB-468 and (b) MCF-10A cells

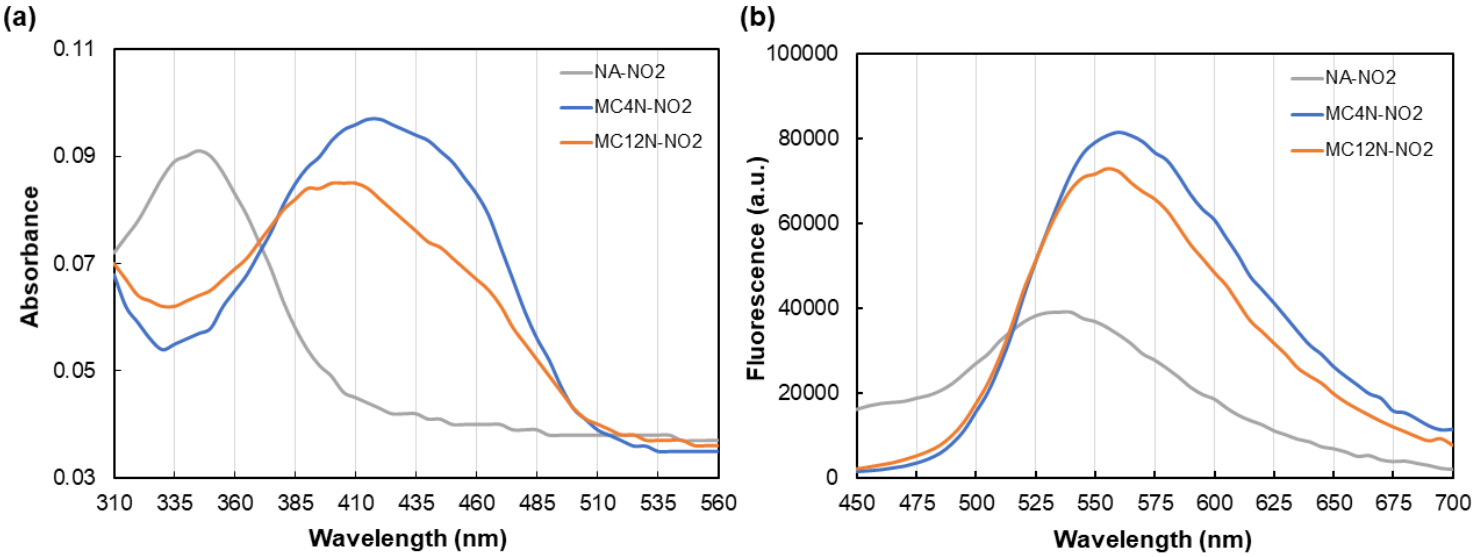

FIGURE S6 (a) Absorption and (b) emission spectra of 4-nitro-1,8-naphthalic anhydride (NA-NO2) and MC4N-NO2 and MC12N-NO2 aptamer-amphiphiles

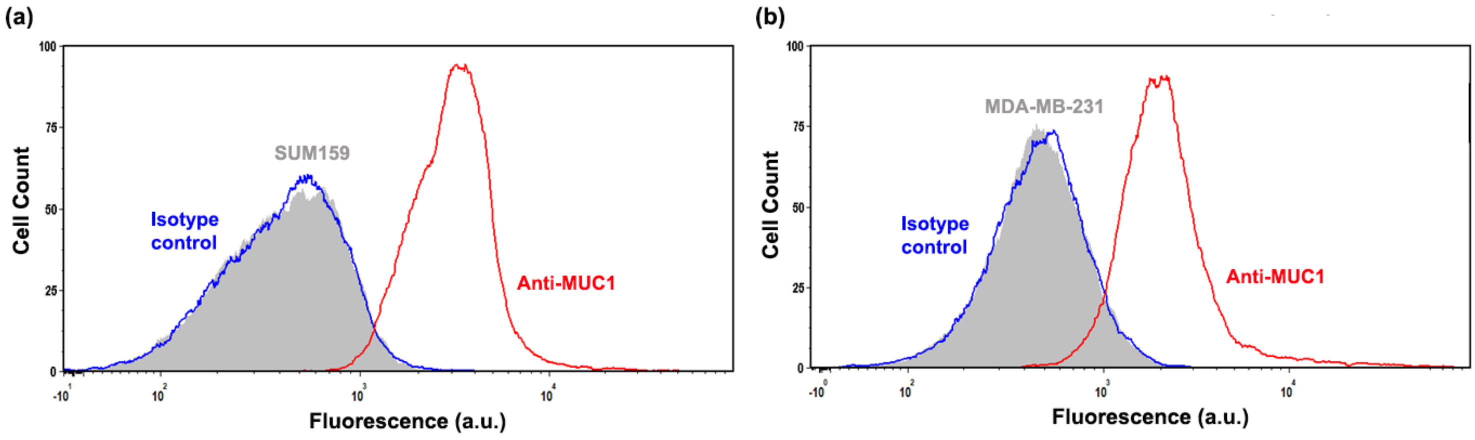

FIGURE S7 MUC1 expression on (a) SUM159 and (b) MDA-MB-231 cells
